# Detecting Seafloor Gas Leakage from Geologic Carbon Storage Sites: A Laboratory-Scale Distributed Acoustic Sensing Evaluation

**DOI:** 10.3390/s26185796

**Published:** 2026-09-12

**Authors:** Brianna C. Miranda, Julia Correa, Jonathan Ajo-Franklin

**Affiliations:** 1 Department of Earth, Environmental & Planetary Sciences, Rice University, Houston, TX 77005, USA; bcm4@rice.edu; 2 Lawrence Berkeley National Laboratory, Berkeley, CA 94720, USA; juliacorrea@lbl.gov

**Keywords:** bubble acoustics (resonance), CO_2_ leakage detection, distributed acoustic sensing (DAS), geologic carbon storage (GCS), variable leakage rate, nearshore environments, passive monitoring, seafloor fiber-optic sensing

## Abstract

Carbon capture and storage (CCS) is a critical technology for mitigating climate change by reducing atmospheric carbon dioxide concentrations. Effective monitoring of CCS sites is essential to ensure that injected CO_2_ remains securely trapped and does not leak into the shallow subsurface or atmosphere. Large-scale CCS in the Gulf of Mexico could be facilitated by extensive existing infrastructure and suitable geologic containment; however, legacy wells and structurally complex geology remain critical challenges for ensuring storage integrity. Traditional monitoring methods, while effective, often lack the temporal resolution and cost-effectiveness needed for comprehensive leak detection, particularly in shallow seafloor environments. This study explores the potential of distributed acoustic sensing (DAS) as a novel monitoring solution for near-surface CO_2_ leakage at geologic carbon storage (GCS) sites. We conducted a laboratory-scale controlled nitrogen gas bubble injection experiment to compare DAS responses at varying cable burial depths and evaluated these signals using simultaneous hydrophone measurements. Results indicate that while DAS effectively detects acoustic signals from bubbles in both sediment and water columns, its response amplitude diminishes with increased burial depth. These findings suggest that DAS could serve as a promising technology for long-term monitoring of marine GCS sites, providing insights into near-surface gas flow and leak detection.

## 1. Introduction

Carbon capture and storage (CCS) is a climate-mitigation approach designed to reduce greenhouse gas concentrations by capturing carbon dioxide from industrial or atmospheric sources and permanently storing it in geologic formations [1]. Monitoring of the storage site is a significant component of CCS and ensures that the injected CO_2_ does not escape the reservoir and re-enter the atmosphere. The Gulf of Mexico has emerged as a focal region for large-scale CCS development in the United States because of its extensive offshore infrastructure, reservoir confinement potential, and proximity to major industrial emission sources. In this region, depleted hydrocarbon reservoirs offer candidate locations for CCS; however, undocumented legacy and orphaned wells raise concerns about potential leakage pathways [2,3]. Additionally, the complex fault geometry of Gulf reservoirs may compromise storage integrity in some cases, as CO_2_ injection could trigger the reactivation of nearby faults [4]. A suite of technologies will be required for the long-term monitoring of these locations with sufficient sensitivity and areal coverage to detect potential CO_2_ leakage from subsurface plumes. Spatially, these technologies must address both subsurface CO_2_ migration at reservoir depths as well as shallower manifestations of leakage in near-seafloor or shallow water environments.

While a variety of techniques are currently utilized for monitoring geologic carbon storage (GCS) sites, many deploy timelapse reflection (4D) seismic surveys or vertical seismic profiling (VSP) in the near-offshore environment [5,6]. These surveys can potentially provide high-quality images of subsurface supercritical CO_2_ (scCO_2_) migration; however, they are costly and often limited by low temporal resolution [7]. Typical survey cadences are annual to less frequent, limiting rapid response to changes in CO_2_ containment. Traditional 4D surveys may also have difficulty in constraining the shallow subsurface and, thus, near–seafloor leakage phenomena. This poses a challenge for identifying gas leaks, which may occur without detection. Large-scale deployment of carbon storage in the near-offshore environment will require monitoring technologies capable of imaging shallow subsurface gas migration and seafloor leakage across extensive areas. For leakage detection, highly repeatable timelapse imaging with high spatial resolution is ideal for identifying gas migration.

Numerous studies have applied distributed acoustic sensing (DAS) VSP to timelapse monitoring of carbon storage. DAS has been utilized in fiber-optic instrumented wells to monitor a 15 kt scCO_2_ injection, observing increases in strain amplitude and travel-time shifts that enabled estimates of plume thickness and reductions in P-wave velocity [8]. These in-well DAS deployments provide dense spatial sampling and continuous measurements along the borehole, allowing for detailed characterization of reservoir-scale CO_2_ migration and associated geomechanical changes. Similar capabilities have been demonstrated at the Aquistore CO_2_ storage site, where DAS-based VSP surveys achieved imaging performance comparable to conventional geophone arrays while offering increased acquisition flexibility and reduced operational costs [9]. DAS has also been integrated into broader measurement, monitoring, and verification frameworks for CCS. In the Minami-aga CCS pilot project, permanently installed fiber-optic cables were used for a range of applications, including VSP, cross-well seismic imaging, microseismic monitoring, and injection profiling, enabling continuous and distributed observation of subsurface processes throughout the injection life cycle [10,11]. More recently, time-lapse crosshole monitoring with downhole DAS detected and tracked velocity anomalies associated with a controlled shallow CO_2_ injection and subsequent upward migration [12].

In addition to borehole deployments, surface-based DAS configurations have also been investigated. Passive monitoring approaches using DAS have emerged as a promising alternative, offering continuous measurements over large spatial extents permitting characterization of the near-surface [13,14]. However, most existing applications focus on active-source seismic imaging or well-coupled reservoir monitoring, whereas the use of DAS for passive detection of near-surface leakage processes remains comparatively underexplored. A recent synthetic feasibility study further explored this application, showing that passive seafloor DAS may detect modeled shallow CO_2_ leakage through changes in ambient noise derived Scholte wave dispersion [15].

Gas bubbles generate passive acoustic signals through radial oscillations at their natural resonance frequency, which is primarily controlled by bubble size, gas thermophysical properties, and the properties of the surrounding fluid [16,17,18]. This relationship forms the basis for interpreting bubble acoustics and relating their spectral properties to gas flux. Passive acoustic monitoring studies have demonstrated that bubble-generated signals can be used to estimate gas flux in controlled environments, where acoustic measurements can be directly related to known injection conditions [19,20]. Additionally, theoretical and experimental studies have established that the spectral characteristics of recorded signals can be used to infer bubble size and emission rates [17,21]. However, these approaches are largely based on hydrophone measurements, and it remains unclear whether DAS exhibits comparable sensitivity to bubble-acoustics or how that sensitivity varies with leakage rate and confining stress.

More recently, lake-floor DAS recorded volcanic degassing in a lake, demonstrating that fiber-optic measurements can capture acoustic signals associated with gas bubble release [22]. While this study supports that DAS can detect bubble-driven acoustic processes, the observations were associated with naturally occurring degassing at low and unconstrained fluxes, and no systematic relationship between gas emission rate and DAS response was established. As a result, although detectability has been demonstrated, the relationship between DAS response amplitude and variations in gas flow remains largely unknown.

To address these uncertainties, we conducted a laboratory-scale controlled gas ebullition experiment to evaluate bubble noise in both the sediment and the water column using DAS and hydrophones. Signal analysis in the time and frequency domains identified bubble resonance signals, compared fiber responses at different burial depths with those in the water column, and tested whether the DAS noise response scaled with gas flow rate. The results confirmed that the hydrophones were highly sensitive to the acoustic wavefield generated by bubbles oscillating in the water column. In comparison, the DAS fiber at the sand–water interface detected signals associated with gas migration through the sediment as well as bubble oscillations in the water column. However, the DAS response amplitude associated with oscillating bubbles rapidly decreased with increasing burial depth. These findings demonstrate that DAS provides a promising technology for long-term monitoring of marine GCS sites, but that the existing fiber geometry or installation strategy should be carefully considered.

## 2. Theoretical Background

### 2.1. Fundamentals of Distributed Acoustic Sensing

Localized perturbations of the fiber produce a change in the phase angle of the Rayleigh backscattered light from pulses at fixed positions ϕ(x,t) separated by a gauge length ($L_{G}$) along the cable. Among conventional interrogator units (IU), this response can be expressed as strain, ε:

(1) $$\varepsilon \left(x,t\right)=\frac{\lambda \;\phi (x,t)}{4\pi nL_{G}\;\xi }$$

where λ is the wavelength, ξ is the photo-elastic constant, and *n* is the refractive index of the fiber. DAS systems use coherent optical reflectometry methods that require lasers with high temporal coherence. If the laser coherence is low, extra phase noise is introduced, which reduces the signal-to-noise ratio (SNR) of the IU [23].

In this study, DAS data were recorded using a modern interrogator unit (Treble, Terra 15) that operates in deformation rate (particle velocity) as its native measurement unit and targets a low instrument noise floor in the high frequency range. This IU is similar to most interrogator units in that it utilizes Rayleigh backscattering to quantify axial strain over a defined gauge length, thereby functioning as a densely sampled sensor array [24,25]. However, it is novel in its method of obtaining the phase measurement. Rather than deriving the phase from the interference of light pulses separated spatially along the fiber, the Treble IU calculates the phase difference between two pulses separated in time. As a result, the Treble IU records a deformation rate quantity that is proportional to the spatial integral of particle velocity along the fiber between the interrogator and each backscatter location, rather than local strain over a fixed gauge length [23]. In this study, a spatial bandpass filter is applied to convert the integrated velocity into local particle velocity. The integrated velocity is expressed as [23]

(2) $$v\left(x,t\right)=\frac{\lambda \;\phi }{4\pi n\Delta t\;\xi }.$$

The optical configuration of this phase-based system is engineered to suppress both amplitude and polarization fading [26], enabling high temporal and spatial resolution suitable for detecting bubble ebullition and other localized high-frequency signals in shallow nearshore environments.

Marine DAS may offer a cost-effective approach for monitoring gas leakage at marine GCS sites due to requiring only a one-time deployment or use of existing seafloor cables, resilience in marine environments, and high spatiotemporal resolution [27]. Real-time imaging or passive monitoring with DAS would also allow for immediate detection of gas leakage, enabling a more rapid response than the annual deployment of ocean bottom seismometers, hydrophones, or campaign surveys. Here, we analyze DAS performance in a controlled environment to assess its efficacy in measuring the geophysical signature of gas bubbles in unconsolidated sediment and free fluid.

### 2.2. Geophysical Signature of Gas Leakage

As long as CO_2_ leakage remains confined within the subsurface, remediation options are limited and the immediate risk to the surrounding environment is minimal. However, once migration pathways extend toward the seafloor, the potential for environmental impact increases substantially. Our zone of interest for monitoring is therefore the shallow marine environment and seafloor interface, particularly the unconsolidated layers that more easily permit gas flow, as well as the water column where bubbles can oscillate freely. We focus on this zone in our experiments because migration to this level marks the stage at which environmental risks escalate and secondary remediation efforts might be considered. Detecting leakage at this stage, however, is challenging because small-scale heterogeneities such as gas bubbles often produce faint seismic diffractions that are difficult for conventional methods to image. As demonstrated in [22], DAS can be sensitive to these anomalies in favorable environments.

**Figure 1:**
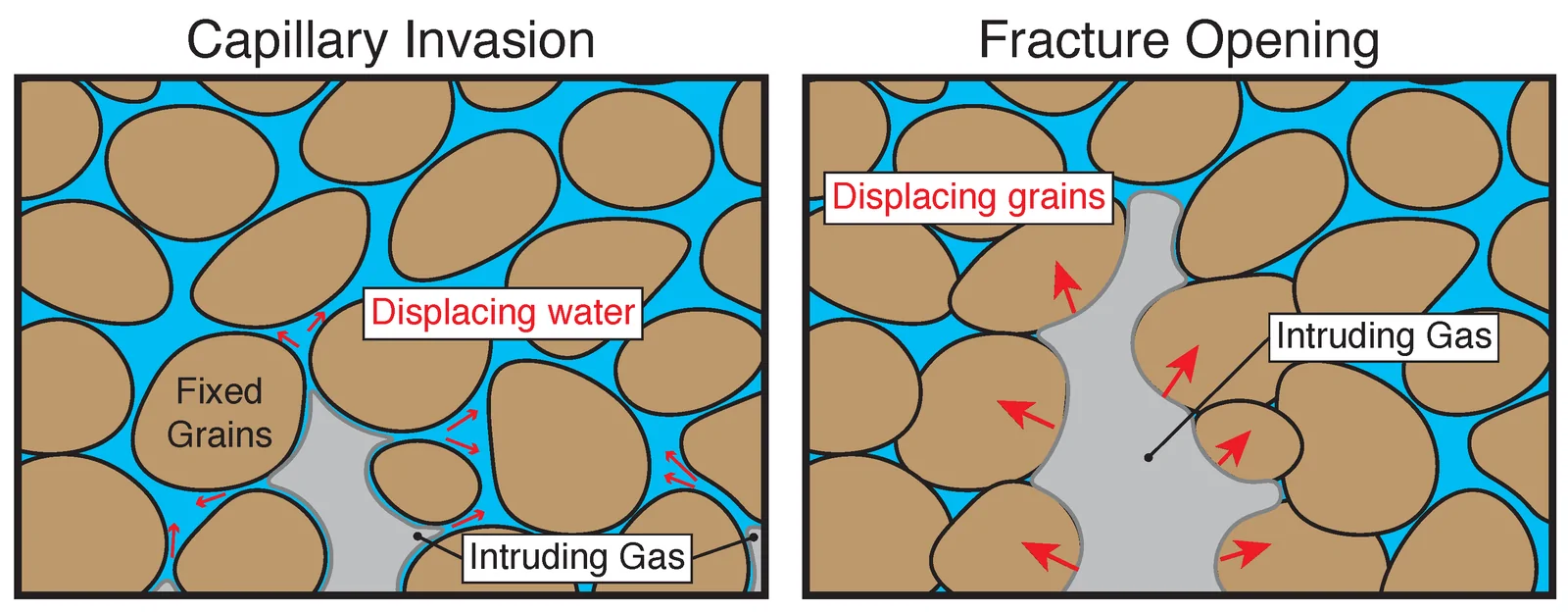
Schematic illustration of gas migration mechanisms in near-surface sediments. (**Left**) Capillary invasion in coarse-grained, consolidated sediments, where large pore throats reduce the capillary entry pressure. In this case, infiltrating gas displaces pore fluids while grains remain stationary. (**Right**) Fracture opening in fine-grained, unconsolidated sediments, where weak grain contacts and elevated capillary entry pressures make it easier for gas to overcome the horizontal stress. Under these conditions, gas displaces grains, leading to fracture formation and bubble generation. Red arrows indicate the displaced component and direction of motion.

The geophysical signature of gas leakage in unconsolidated sediments is governed by how gas migrates through pore networks under conditions set by both sediment strength and the prevailing in-situ stress field. Migration may occur by (1) displacement of pore fluids once the capillary threshold pressure of the pore network is exceeded or (2) displacement of the grains themselves and deformation of the sediment. The first process, known as capillary invasion, typically dominates in consolidated, coarse-grained sediments at greater burial depths, where large pore throats lower the capillary entry pressure and enable gas to infiltrate the pore space (Figure 1). In contrast, shallow fine-grained sediments are weakly compacted and characterized by small pore throats, resulting in high capillary entry pressures. Because the grains are poorly consolidated, the stress required to displace them is lower than the pressure needed to overcome capillary entry. Under these conditions, gas migration proceeds primarily through grain displacement and fracture opening rather than pore infiltration. In marine unconsolidated sediments, fracture opening is generally the dominant mechanism, although capillary invasion may still act as a precursor depending on the degree of compaction. As migrating gas accumulates, it exerts pressure on the surrounding grains, causing them to deform elastically and form localized pockets via fracture opening [28,29]. When the buoyancy of these pockets exceeds the confining stress, discrete bubbles detach and ascend vertically through the sediment [28].

When a gas bubble emerges from the seafloor into the water column, it oscillates freely in a manner analogous to a mass on a spring. At small amplitudes, this motion can be described as simple harmonic oscillation occurring at the bubble’s natural, or resonance, frequency [16]. The resonance frequency is defined by Minnaert’s equation:

(3) $$f=\frac{1}{2\pi r}\sqrt{\frac{3\kappa p_{0}}{\rho }}$$

where *r* is the bubble radius, κ is the polytropic index of the gas, $p_{0}$ is the hydrostatic pressure at the bubble’s depth, and ρ is the density of the surrounding fluid [16]. Because this resonance characterizes the passive acoustic signature of bubbles, we hypothesize that it also influences their geophysical signature. In this study, the Minnaert equation is applied to compare DAS and hydrophone responses to bubble size and leakage rate, thereby assessing the sensitivity of DAS to gas ebullition in unconsolidated sediments and free fluid.

## 3. Materials and Methods

### 3.1. Experimental Setup

To evaluate the effectiveness of seafloor distributed acoustic sensing (DAS) for gas leakage detection, we designed and constructed a laboratory-scale testing environment that isolates bubble acoustics in a controlled setting. This configuration minimizes competing noise sources common in field data and allows for direct analysis of the DAS response to gas leakage. The experiment was designed to allow accurate manipulation of gas flow rates while recording passive signals at a range of DAS burial depths.

**Figure 2:**
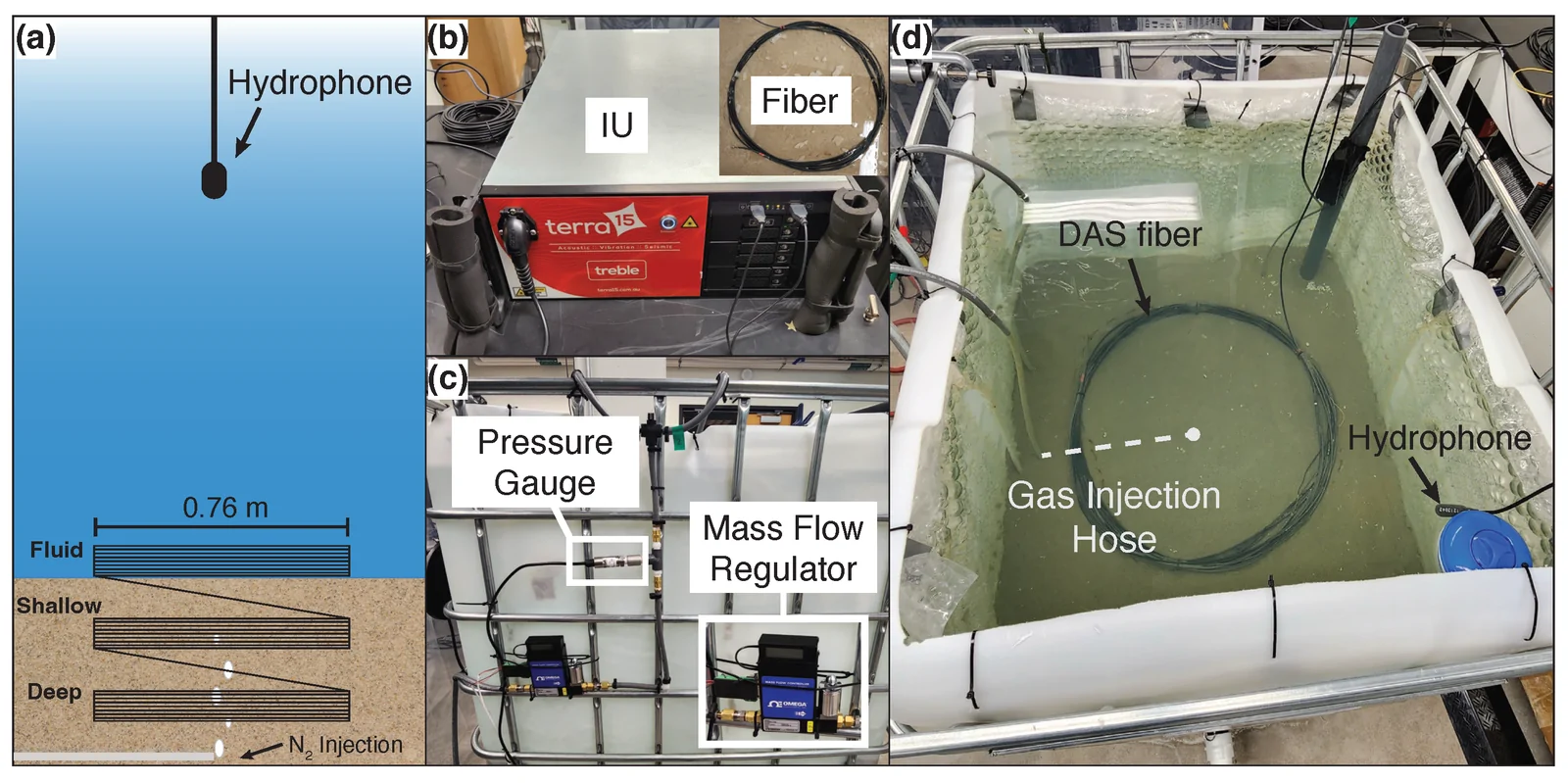
Setup and components of the laboratory-scale gas injection experiments. (**a**) Cross-sectional schematic of the experimental design, showing the distributed acoustic sensing (DAS) fiber coiled around a mesh frame in three layers: one in the fluid above the sand, one shallowly buried 5 cm below the sand–water interface, and one buried at 18 cm depth. Each layer represents 20 m of fiber. (**b**) DAS interrogator unit (Treble, Terra 15), which records data on the fiber coil (unit shown with coiled fiber). (**c**) Mass flow regulator used to regulate gas injection rates. (**d**) Photograph of the assembled testing environment. The reference hydrophone is visible near the water surface and slightly off-center for illustration, but during testing it was positioned 60 cm above the gas injection point.

The testing environment consisted of a 1250-liter intermediate bulk container (IBC) tank with internal dimensions of 1.0 m × 1.2 m × 1.35 m. To reduce acoustic reflections during experiments, the tank walls were lined with closed-cell plastic bubble wrap; however, this was intended to reduce rather than eliminate boundary reflections. A layer of fine-grained 40/70-mesh sand was placed at the base of the tank to simulate unconsolidated marine seafloor sediments. The DAS fiber (single-mode tactical fiber) was coiled around a wire mesh in three layers, each corresponding to 20 m of fiber, which is the minimum length required for reliable acoustic measurements. The total fiber length was 120 m, and the coiled mesh had a diameter of 76 cm. Two fiber layers were buried at different depths within the sand (deep and shallow placements), while the third was positioned above the sand–water interface (Figure 2a).

While DAS measurements were acquired with the IU (Figure 2b), a hydrophone (HTI-96-MIN, High Tech, Inc., Long View, MS, USA), suspended approximately 60 cm above the sand–water interface and centered above the gas injection point, provided simultaneous control measurements (Figure 2a), shown off-center in Figure 2d. The gas injection hose was installed at the base of the sand layer, directly in the center of the fiber coil (Figure 2a,d). For safety, nitrogen gas was used as a surrogate for CO_2_ to avoid risks associated with confined indoor CO_2_ injection. Flow rates were controlled using a mass flow regulator (model FMA5518A, 0–5 L min^−1^ N_2_; OMEGA Engineering, Inc., Norwalk, CT, USA) (Figure 2c). Experimental injection rates are summarized in Table 1.

### 3.2. Experimental Procedures

Experiments performed in the testing tank included a baseline acquisition, a controlled bubble release recorded by hydrophone only, injections at multiple flow rates, and a “single bubble” release. A hydrophone recorded data simultaneously with the DAS IU and was used as a control measurement because it is known for its ability to accurately record the acoustic properties of bubbles in the water column. To characterize the spectral properties of bubble oscillations, a preliminary start/stop experiment was conducted using only the hydrophone for data acquisition. This was performed at a flow rate of 600 mL min^−1^. This flow rate was sufficiently high to produce continuous bubble emergence from the sand-water interface and was therefore expected to produce an identifiable spectral response. In this experiment, bubble injection was delayed by 10 s at the onset of recording, released for 30 s, then terminated 10 s prior to the end of the recording.

For injection experiments, DAS and hydrophone responses were recorded simultaneously for flow rates from 100 to 1000 standard mL min^−1^ N_2_ in 50 standard mL min^−1^ increments. At equivalent mass flux, these N_2_ injection rates correspond to approximate CO_2_ leakage rates of ~0.066–0.657 tonnes yr^−1^. These CO_2_-equivalent rates represent mass flux scaling only and do not imply identical bubble dynamics between N_2_ and CO_2_, which also depend on gas-specific thermophysical properties [16,17,18]. In the context of geologic carbon storage, these rates represent very small leakage rates; for example, the Sleipner project in the North Sea has injected on the order of 1 Mt yr^−1^ [30]. Bubble release data were recorded five times at each flow rate for 30 s to assess variability. A final low-rate experiment at 50 mL min^−1^ was intended to isolate the acoustic signature of a single bubble. However, even at this lowest flow rate the injection produced a small cluster of bubbles rather than a single discrete event. Because continuous injections consistently yielded overlapping emissions that merged acoustically, we adopt the term *bubble cluster* to describe these short intervals of oscillatory energy with clear spectral concentration. Bubble clusters serve as the practical observational unit in our experiments: they emulate the waveform morphology of single bubbles but reflect the overlapping release dynamics and tank reverberation present during continuous injections. While isolated single-bubble emissions are often emphasized in seep studies [22], sustained or episodic leakage from compromised wells or fractures is more likely to manifest as repeated or clustered bubble release. As such, the bubble clusters observed in this study represent a realistic acoustic expression of continuous or intermittently venting leakage pathways. Injection pressure data were also acquired (PX309 Series, OMEGA Engineering, Inc., Norwalk, CT, USA) and are available in the Supplementary Materials (see Figure S1).

Bubble dimensions were estimated from still frames extracted from camera footage (GoPro) acquired during bubble release events. Measurements of the bubble major and minor axes were obtained using a calibrated grid backdrop, from which an equivalent spherical radius was calculated. These equivalent radii were used to estimate bubble resonance frequencies using the Minnaert relation (Equation (3)). As the analysis is based on still-frame imagery rather than time-resolved oscillatory measurements, the calculated frequencies represent theoretical resonance estimates.

### 3.3. Data Processing

DAS recordings were processed to isolate bubble-related acoustic signals through conversion of the raw measurements and the application of successive filtering and spectral analyses. DAS data were acquired with a gauge length of 0.8168 m, a pulse length of 0.8168 m, a pulse rate of 284.09 kHz, and a temporal sampling rate of 2.2 kHz. As previously stated, the DAS IU data are natively recorded as integrated velocity and were converted to local velocity prior to preprocessing using a first-order spatial bandpass filter. The filter used a cutoff wavelength of $L_{0}=100$ m, with $k_{0}=2\pi /L_{0}$ and a spatial sampling interval of Δx=0.8168 m. The spatial filter, defined by the transfer function $H\left(z\right)=\left(1-z^{-1}\right)/\left[\left(1+k_{0}\Delta x\right)-z^{-1}\right]$, was applied in the forward and reverse directions along the spatial channel dimension. The local velocity-converted data were divided into three segments corresponding to the fiber lengths associated with the deep sand, shallow sand, and fluid layers. Each segment then underwent de-meaning, de-trending, and highpass filtering at 1.5 Hz, with a Hann taper applied prior to spectral analysis. A short-time Fourier transform was applied to produce spectrograms using 0.09 s (198-sample) windows with 90% overlap and an fast Fourier transform (FFT) length of 131,072 samples. The resulting spectrograms were subsequently smoothed using a three-channel spatial median filter to reduce noise. Amplitude spectra were obtained from the local velocity data using an FFT transform and were used to determine bubble bandwidth. Single-sided amplitude spectra were calculated using the full analysis record with no zero-padding. FFT amplitudes were normalized by the number of temporal samples, with positive-frequency amplitudes doubled except at 0 Hz and the Nyquist frequency. The frequency resolution was $\Delta f=f_{s}/N$, where $f_{s}=2.2$ kHz and *N* is the number of samples in the analyzed record. Amplitude spectra were calculated independently for each DAS channel and summed across channels within each fiber segment to obtain the segment-level spectral response. Identical filtering and spectral-processing parameters were applied to all fiber segments. A five-point moving average filter was applied to the amplitude spectra, and the root mean square (RMS) amplitude was calculated to evaluate trends with flow rate and burial depth. For DAS, RMS amplitude was calculated over the full time series of each channel, averaged across channels within each fiber segment, and subsequently averaged across the five repeated injections at each flow rate. Hydrophone RMS amplitudes were similarly calculated for each processed pressure record and averaged across the five repeated injections.

Hydrophone data underwent similar processing but required additional filtering due to their lower SNR (Figure S2). Initial steps included demeaning and detrending, followed by a bandpass filter with linear tapers from 1.5–30 Hz and 1800–1900 Hz, and the passband from 30–1800 Hz. A notch filter was applied to baseline data to remove 60 Hz electrical noise, and a highpass filter at 10 Hz was used to eliminate low-frequency artifacts generated by the acquisition device. As with the DAS data, a Hann taper was applied prior to spectral analysis.

For qualitative waveform and spectrogram interpretation, representative bubble cluster intervals were identified visually where oscillatory waveforms and concentrated bubble-band energy were evident. The quantitative RMS analysis and flow rate regressions were calculated independently from the complete processed records and therefore did not depend on manually selected cluster boundaries.

## 4. Results

### 4.1. Hydrophone Response to Bubbles in Fluid

**Figure 3:**
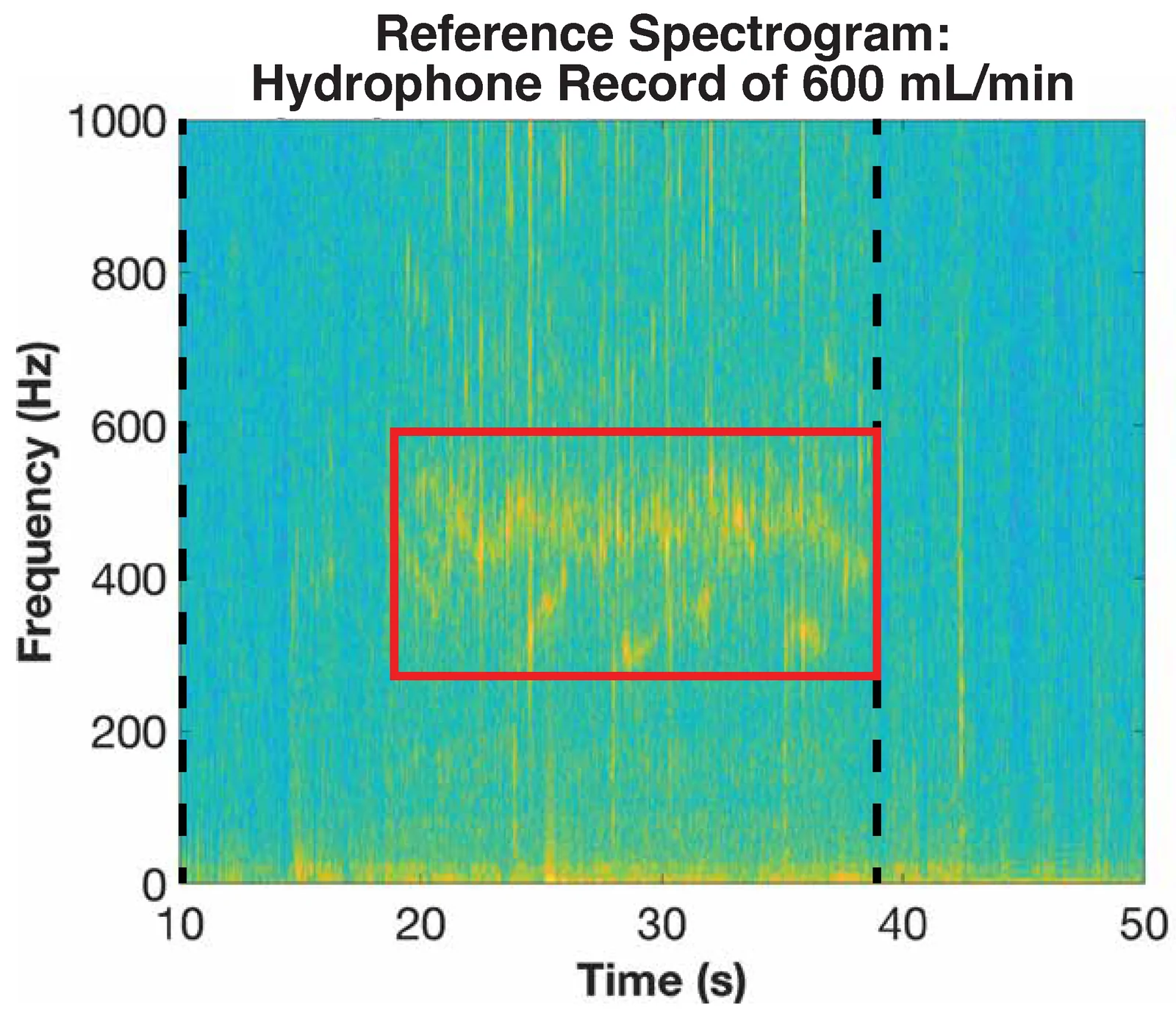
Power spectral density identification of nitrogen gas bubbles in the water column from preliminary tests with a hydrophone. Nitrogen gas injection begins at 10 s and terminates at 39 s, as indicated by the black dashed lines. Bubble emergence begins at 19 s and ends with the termination of the injection, with the spectral traces corresponding to the bubbles outlined by the red box. The average bandwidth of the bubbles is 250–600 Hz.

The time–frequency spectra for the hydrophone measurement at 600 mL min^−1^ revealed a distinct cluster of high spectral density between 19 s and 39 s (outlined in red in Figure 3). The gas injection began at 10 s; however, spectral signatures are only visible starting at 19 s. This delay reflects the time required for the buoyancy force of the gas to overcome both the effective stress of the overburden and the capillary entry pressure of the pore space, conditions necessary for fracture opening and vertical migration of the bubble into the water column. The spectral signatures observed in the hydrophone record are therefore attributed to bubbles oscillating in the water column. The continuous bubble stream frequency band falls predominantly between 250–600 Hz; hence, the DAS data should exhibit a similar spectral response within this frequency range.

### 4.2. DAS Response to Gas in Fluid and Sediment

**Figure 4:**
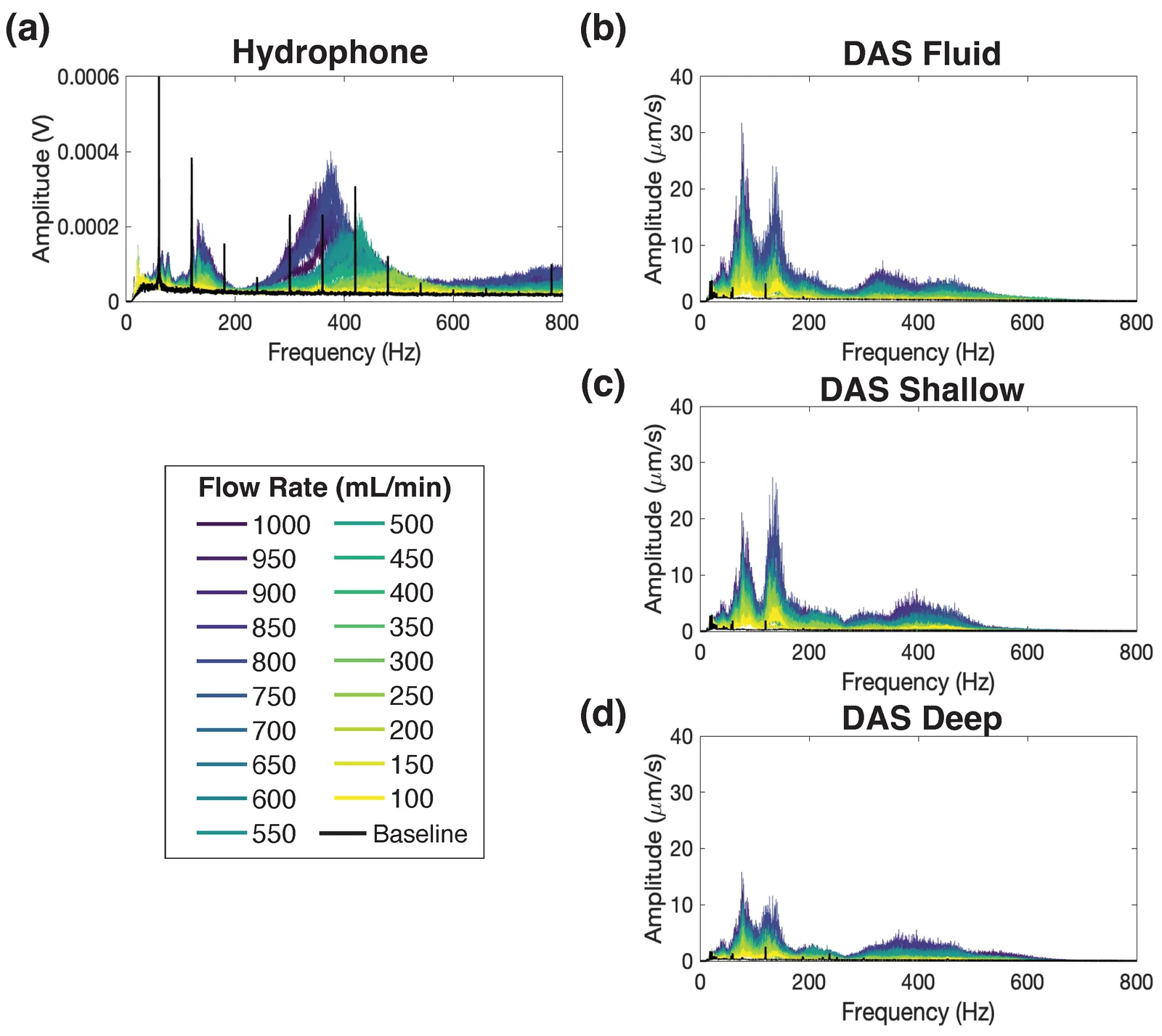
Amplitude spectra of bubble cluster acoustic signals recorded by the hydrophone and DAS at varying burial depths across multiple injection flow rates. (**a**) Hydrophone amplitude spectra, showing a dominant bubble resonance band between approximately 270 and 600 Hz. (**b**) DAS fluid-layer fiber amplitude spectra, which resolve the same bubble resonance band and additionally exhibit two distinct low-frequency peaks centered near 77 Hz and 135 Hz. (**c**) DAS shallow-buried fiber spectra, showing attenuation of the low-frequency peaks relative to the fluid layer while maintaining the resonance band. (**d**) DAS deep-buried fiber spectra, where low-frequency amplitudes are further attenuated with increasing burial depth. Across all panels, colored traces correspond to increasing injection flow rates, and the baseline spectrum is shown for reference.

The analysis of the amplitude spectra derived from the DAS data focused on each injection rate to characterize the bubble resonance and the associated low-frequency signals. In the analyses that follow, bubble clusters are treated as the fundamental observational unit. Observations indicated that bubble resonance occurred within a bandwidth of approximately 270–600 Hz (Figure 4b), which corresponds with measurements taken from hydrophones. However, the DAS fluid layer, which is most comparable to the hydrophone measurements, exhibited two distinct low-frequency amplitude peaks centered around 77 Hz and 135 Hz (Figure 4b). These peaks were also observed in the sand-coupled fiber layers (Figure 4c,d) and in the hydrophone response, although they were less prominent in the hydrophone spectrum than in the DAS spectra.

The vertical distribution of the fiber enabled a depth-dependent analysis of the DAS response. Examining the three burial depths showed that the amplitude of the low-frequency peaks attenuated with increasing distance from the sand–water interface (Figure 4b–d). The fluid-layer segment of the fiber exhibited the largest spectral amplitudes in both the bubble resonance band and the low-frequency peaks, while the shallow and deep layers exhibited progressively weaker responses. In comparison, the resonance band maintained relatively stable amplitudes, showing only small frequency shifts across layers.

**Figure 5:**
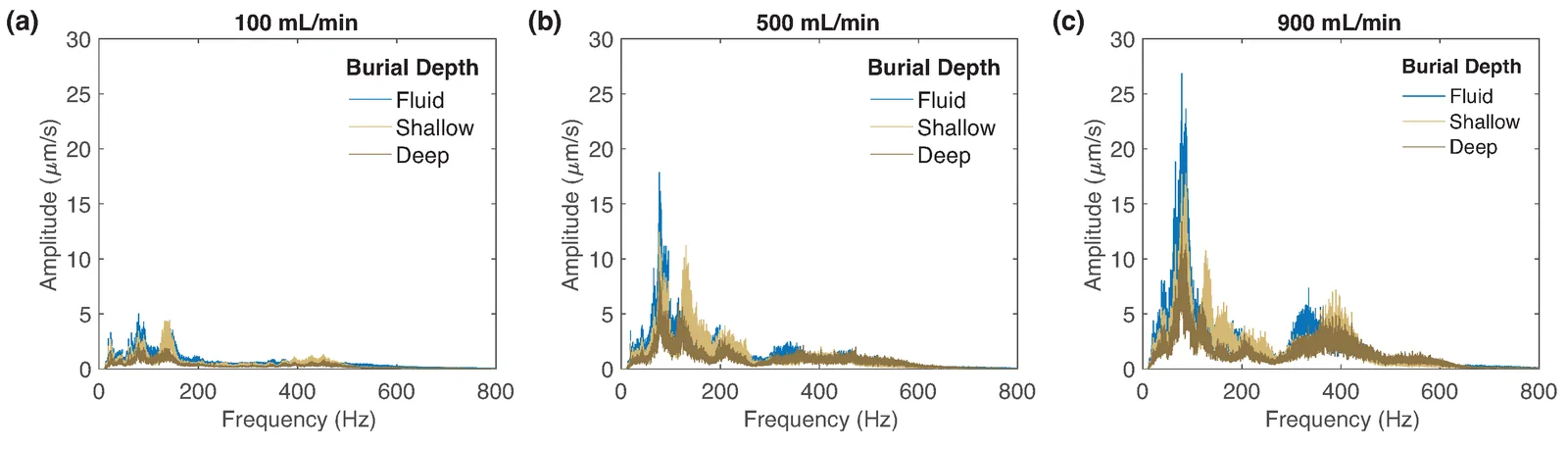
Amplitude spectra of DAS measurements at three representative injection flow rates illustrating flow rate dependence across fiber burial depths. Panels show spectra for (**a**) 100 mL min^−1^, (**b**) 500 mL min^−1^, and (**c**) 900 mL min^−1^ injections, with responses from the fluid-layer, shallow-buried, and deep-buried fibers plotted in each panel. Increasing flow rate results in higher spectral amplitudes across all burial depths, accompanied by enhanced low-frequency energy and broadening of the bubble resonance band. Attenuation of spectral amplitude with increasing burial depth is consistently observed at all flow rates.

The DAS response exhibited a dependence on gas injection rate. Representative amplitude spectra for flow rates of 100, 500, and 900 mL min^−1^ are shown in Figure 5a–c for the fluid-layer, shallow-buried, and deep-buried fiber segments. Across all burial depths, increasing flow rate is associated with higher spectral amplitudes within the bubble resonance band and increased prominence of low-frequency energy. Between 100 and 500 mL min^−1^, substantial broadening of the resonance bandwidth is observed, indicating an increasing spread of dominant frequencies with flow rate. This behavior may reflect greater variability in bubble radii at higher injection rates, consistent with the Minnaert relation (see Equation (3)). At 900 mL min^−1^, both the resonance band and low-frequency components exhibit elevated amplitudes across all fiber layers, although attenuation with burial depth remains evident. These observations demonstrate a positive correlation between injection flow rate and the magnitude and spectral extent of DAS-recorded bubble-related acoustic energy.

**Figure 6:**
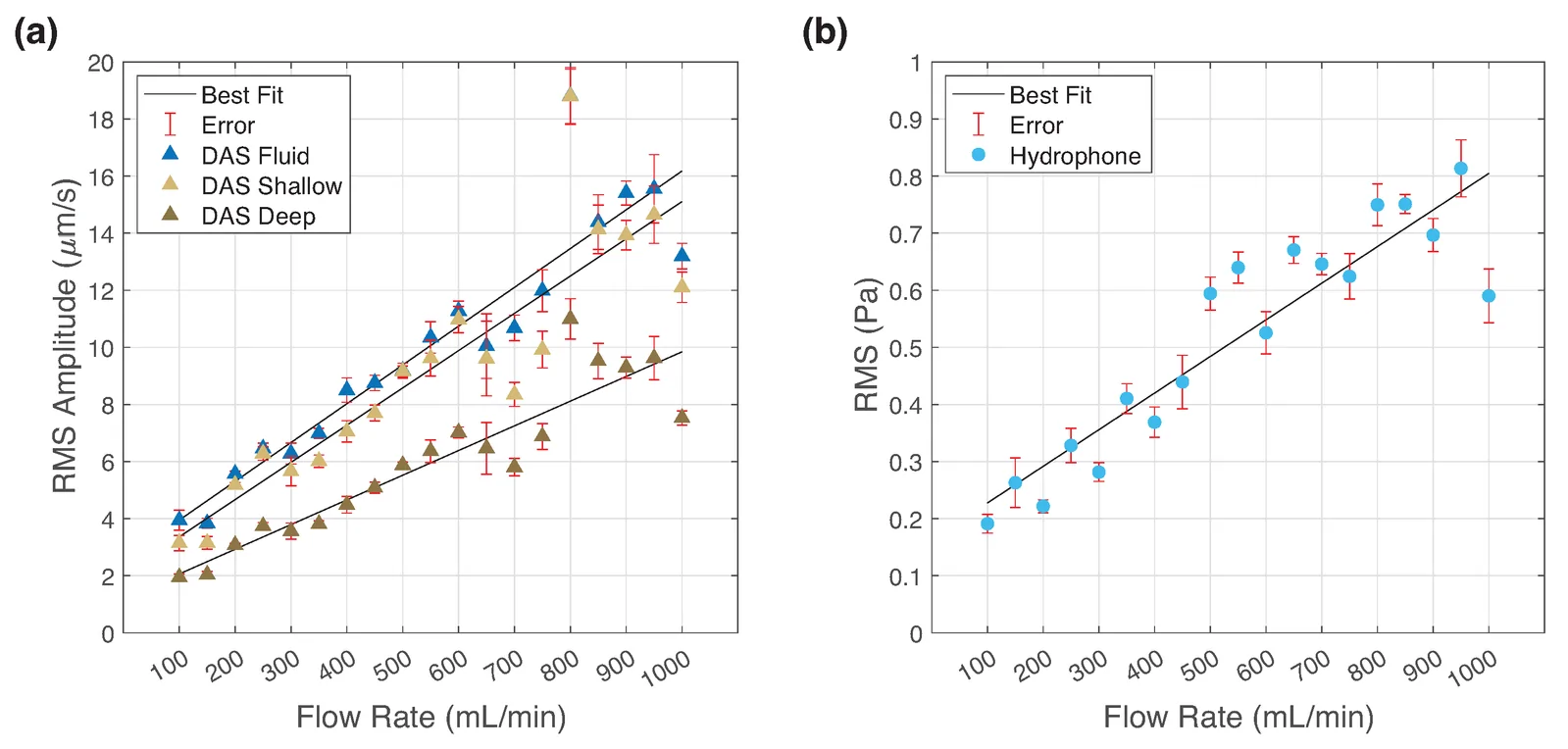
Root mean square (RMS) amplitude of acoustic signals as a function of gas injection flow rate for DAS and hydrophone measurements. (**a**) DAS RMS amplitudes for the fluid-layer fiber, shallow-buried fiber, and deep-buried fiber, with linear best-fit trends shown for each depth. Error bars represent the standard error of the mean across five repeated injections at each flow rate. The deepest buried fiber exhibits a near-linear increase in RMS amplitude with flow rates below approximately 650 mL min^−1^, while the fluid and shallow layers show similar linear trends with increased scatter. (**b**) Hydrophone RMS amplitudes as a function of flow rate, showing flow rate-dependent variability within the hydrophone measurements. Because the hydrophone and DAS measure different physical quantities, their absolute RMS amplitudes are not directly comparable.

The relationship between injection flow rate and acoustic amplitude was quantified using the RMS of the recorded signals (Figure 6a). Because the hydrophone and DAS measure acoustic pressure and local particle velocity, respectively, their absolute RMS magnitudes cannot be directly compared as measures of relative sensitivity. Instead, the RMS response of each sensing system is evaluated independently as a function of injection flow rate. Across the full 100–1000 mL min^−1^ range, linear regressions explained a substantial proportion of the variability in RMS amplitude, with $R^{2}=0.849$, 0.785, and 0.856 for the deep, shallow, and fluid DAS segments, respectively, and $R^{2}=0.843$ for the hydrophone; all regression slopes were statistically significant (p<0.001). The hydrophone measurements exhibit greater variability across the tested flow rates, whereas the DAS response shows a generally increasing RMS trend with flow rate, particularly for the deepest fiber segment up to approximately 650 mL min^−1^. Cross-sensor detection performance is evaluated separately using SNR (Figure S2).

### 4.3. Comparison with the Minnaert Relation

The Minnaert frequency of the bubbles was calculated for both the lowest and highest flow rates (100 mL min^−1^ and 1000 mL min^−1^) using video footage from the gas injection experiment. To do this, the elliptical dimensions of the bubbles, captured in freeze frames, were converted into spherical radii based on the bubble area. This allowed us to compare the measured frequency of the bubbles with their theoretical frequency using Equation (3). The Minnaert frequency is influenced by the bubble’s radius, which is in turn affected by its depth (due to hydrostatic pressure) and the temperature gradient within the water column. In the shallow-water experimental tank, the temperature gradient is negligible; however, the pressure gradient results in an approximate 75 Hz variation in bubble frequency as the bubble rises from the sand-water interface to the surface.

**Figure 7:**
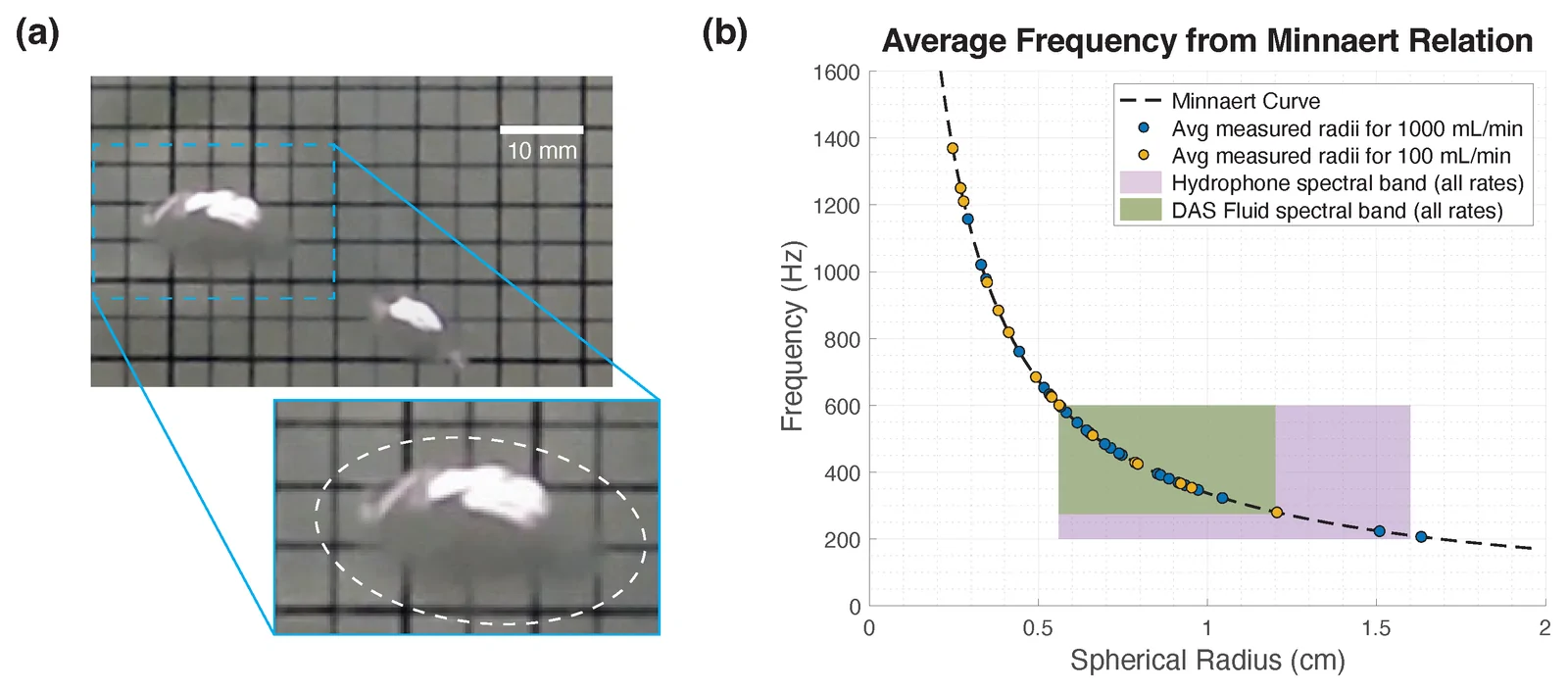
(**a**) Still frame from camera footage showing an ellipsoidal bubble released during injection. Bubble dimensions were approximated from multiple still frames using the grid backdrop, and an equivalent spherical radius was computed from these measurements. The resulting radii were used to estimate bubble resonance frequencies via the Minnaert relation (Equation (3)). (**b**) Measured equivalent spherical radii and their corresponding Minnaert-predicted resonance frequencies. The theoretical frequency–radius relationship is shown by the Minnaert curve (black dashed line). Bubble cluster frequencies derived from camera-based measurements at the minimum (yellow) and maximum (blue) injection flow rates are plotted along this curve, illustrating radius and frequency variability across experimental conditions. Shaded regions denote the frequency bands directly observed in the hydrophone data (purple) and in the DAS measurements from the fluid-layer fiber (green).

To determine an average resonance frequency, we utilized the bubble radii observed at the surface of the tank (as depicted in Figure 7a) along with the estimated bubble radius at the bottom of the tank, which was derived from surface measurements using Boyle’s Law. This approach facilitates a comparison between the frequency estimated from visual observations and the measurements obtained from the DAS and the hydrophone.

Figure 7b presents these results, showing that the theoretical Minnaert frequencies computed from visual observations for the 100 and 1000 mL min^−1^ injections are more broadband, at approximately 200–1400 Hz. The experimental Minnaert frequencies correspond to a spherical bubble radius of 0.6–1.2 cm for the DAS data and 0.6–1.6 cm for the hydrophone data. When we compare these with the observed radii from footage of bubble release at these flow rates, we find that the DAS measurements capture the lower frequency range where the majority of bubbles are present, while the hydrophone response detects a broader lower frequency limit, capturing a few larger bubbles. Minor discrepancies between Minnaert-predicted frequencies and observed acoustic responses may be attributed to uncertainties in radius estimation, including deviations from spherical geometry and radial lens distortion associated with wide-angle camera optics. The comparison between visually derived bubble radii and acoustically observed frequency bands is intended as a first-order consistency check rather than a quantitative validation of Minnaert scaling or of a flow rate dependence in bubble size. Agreement between the theoretical frequency range and the dominant observed resonance bands supports the interpretation that the recorded acoustic energy arises from bubble oscillations, even though precise frequency matching is limited by radial lens distortion, non-spherical geometry, and cluster superposition effects.

**Figure 8:**
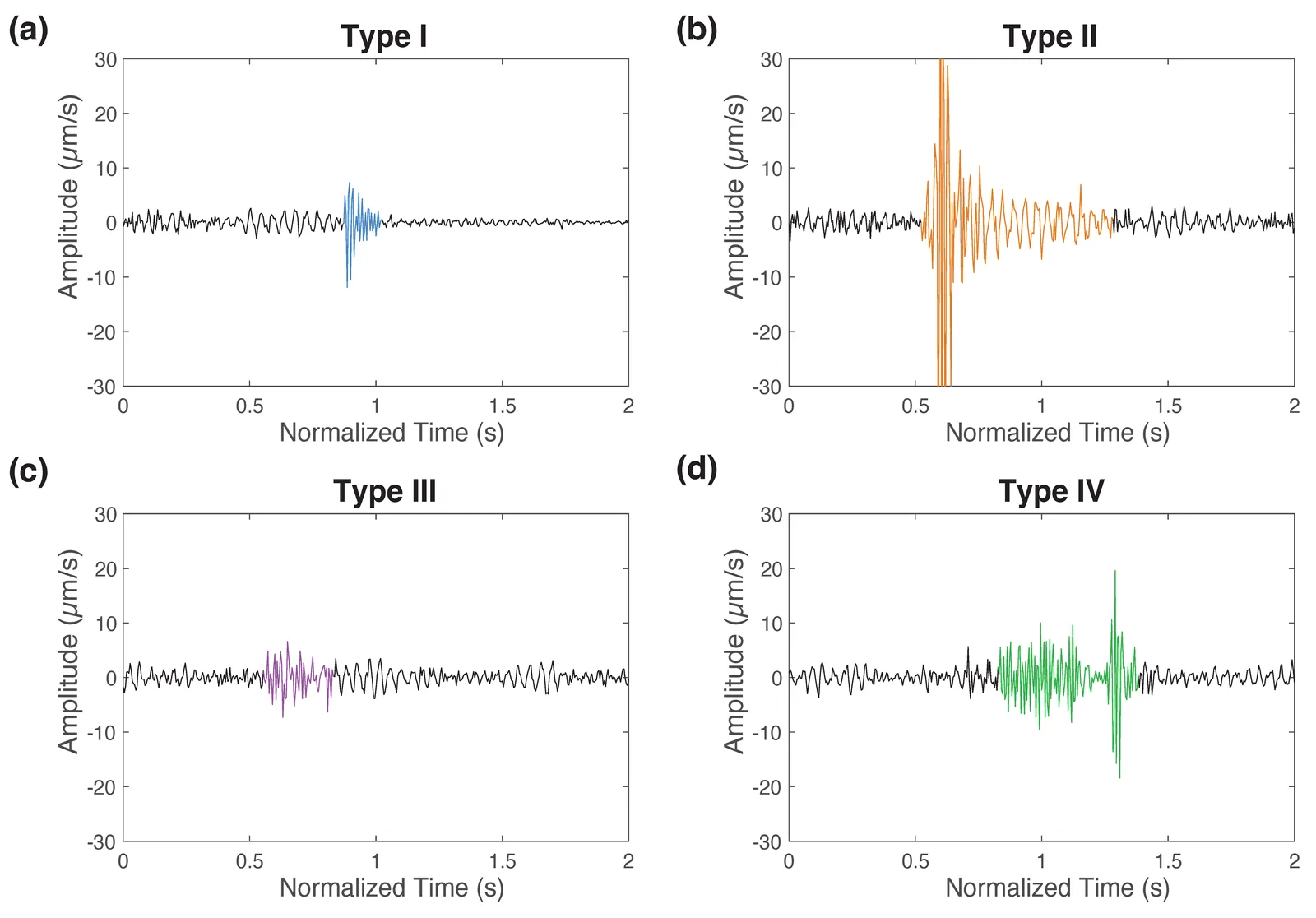
Representative bubble cluster–associated acoustic waveforms categorized into four types (Type I–IV), with color used only to distinguish the highlighted waveform in each panel. (**a**) Type I waveforms exhibit the characteristic oscillatory signature of a relatively isolated bubble in free fluid and serve as the reference case for typical bubble dynamics. (**b**–**d**) In contrast, Type II–IV waveforms deviate from this canonical oscillatory behavior and are interpreted as cluster responses influenced by interactions at the sediment–water interface. These waveforms are hypothesized to reflect interface-controlled mechanisms (e.g., coupling with the sediment boundary and bubble–bubble interactions) rather than the free-floating oscillatory dynamics observed in Type I.

**Figure 9:**
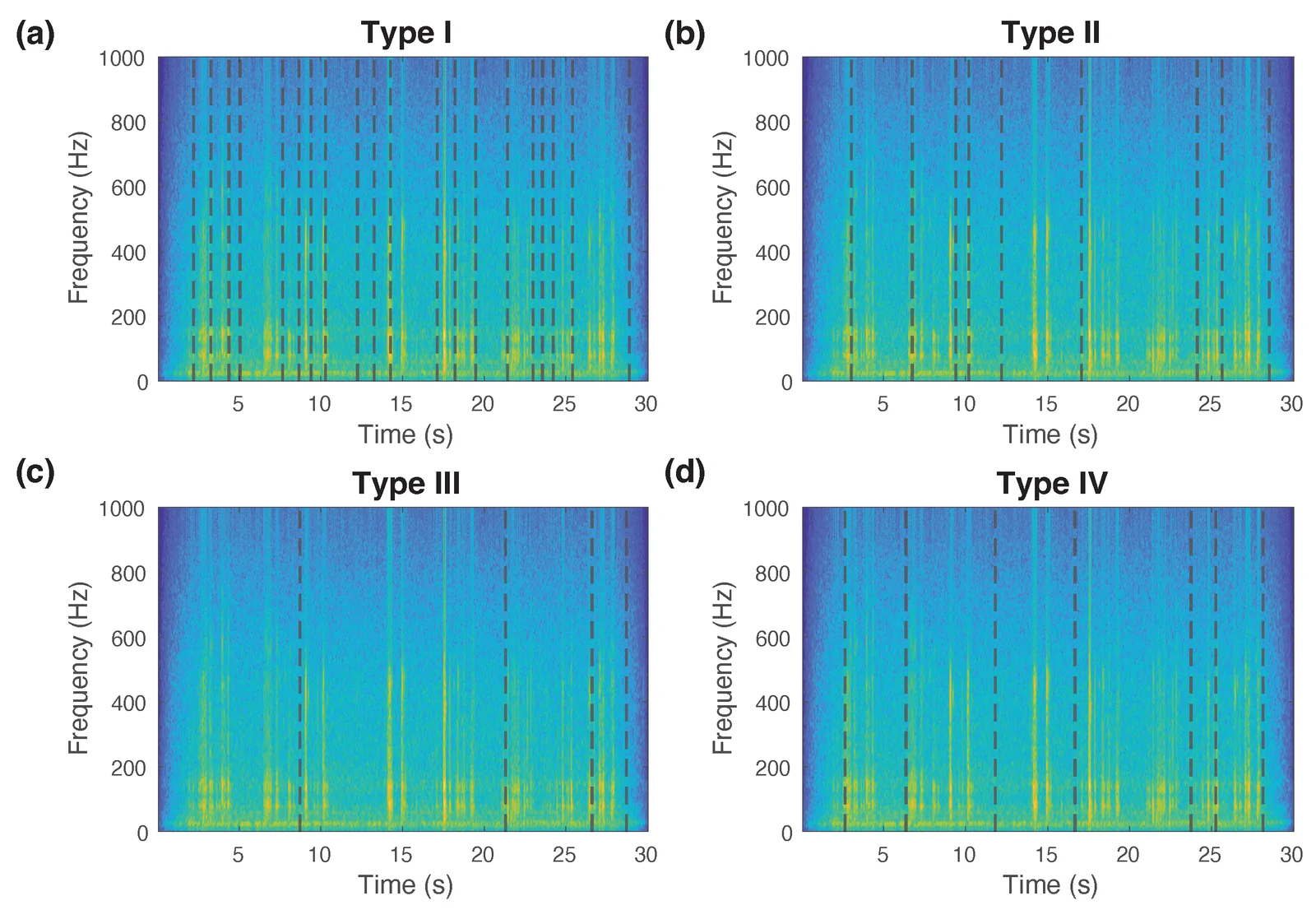
Results of template matching applied to bubble cluster–associated acoustic signals using a normalized cross-correlation approach. Panels (**a**–**d**) correspond to waveform Types I–IV, respectively, and show time–frequency spectrograms for the 100 mL/min injection case. A match is defined as a waveform exhibiting ≥ 49% normalized similarity to the corresponding template. Black dashed lines indicate arrivals satisfying this criterion.

From the low-rate experiment at 50 mL min^−1^, representative bubble-associated waveform templates were identified and cross-correlated with the time-domain acoustic records from each injection rate. Four waveform types were identified within the low-rate dataset (Figure 8). The Type I waveform is associated with a relatively isolated bubble event emerging from the sediment after the injection terminated, whereas Types II–IV occurred during active injection and are associated with cluster-like emergence, with Type II representing the first major cluster to emerge from the sediment–water interface. A normalized cross-correlation threshold of 49% was selected empirically by testing a range of correlation values and identifying the value at which the majority of visually identifiable bubble-associated spectral traces were recovered without extensive overmatching. The same threshold was then applied uniformly across all waveform types and injection rates to avoid template-specific threshold tuning. Figure 9 shows application of the templates derived from the 50 mL min^−1^ experiment to the separate 100 mL min^−1^ injection record. Type I matches generally coincide with prominent spectral traces but also include additional detections where no clearly distinguishable bubble-associated spectral feature is observed. In contrast, Types II–IV yield fewer, more selective matches that are not always perfectly aligned with visually identifiable spectral features.

## 5. Discussion

### 5.1. Interpretation of Bubble Acoustics Measurements

The acoustic emissions recorded in the tank experiments were dominated by quasi-continuous bubble clusters rather than isolated single bubbles. Each cluster represented a rapid succession of overlapping bubble oscillations produced as gas escaped intermittently through the sediment. Although this differs from natural seepage observations emphasizing discrete bubble events, the cluster structure retains the oscillatory morphology and spectral character of individual bubbles. The persistence of a resonance band across clustered emissions indicates that superposition of multiple bubble oscillations does not obscure the characteristic frequency range, supporting cluster-scale spectral interpretation of bubble activity.

The differences between hydrophone and DAS measurements may reflect their respective applicability to various monitoring scales, particularly in terms of spatial coverage and imaging capabilities. Both sensors effectively resolve fundamental bubble resonances between approximately 270 and 600 Hz, although their recorded responses differ because the hydrophone measures localized acoustic pressure, whereas DAS measures distributed fiber motion. Consequently, absolute spectral amplitudes cannot be directly compared between the two systems. The hydrophone response exhibits greater variability, particularly at lower flow rates, whereas the DAS measurements show a relatively stable acoustic response and higher SNR (Figure S2), indicating that bubble-associated signals were more distinguishable above the respective background noise levels in the DAS data under the experimental conditions tested.

Changes in flow rate are correlated with significant alterations in the spectral response, providing insights into the underlying physical processes that affect DAS measurements. Increasing the injection rate from 100 to 600 mL min^−1^ caused a near-linear rise in RMS amplitude ($R^{2}=0.970$, 0.948, and 0.975 for the deep, shallow, and fluid DAS segments, respectively; p<0.001 for all) and a broadening of the resonance bandwidth. The amplitude increase is consistent with greater bubble flux, while the bandwidth expansion may reflect the simultaneous presence of bubbles with varying radii. At the highest rates (≥650 mL min^−1^), the trend became nonlinear and more variable, suggesting the onset of coalescence, overlapping bubble trains, and localized sediment disturbance. These effects likely introduce transient low-frequency energy and modulate the resonance envelope, consistent with the broader DAS spectra observed under those conditions.

### 5.2. Low-Frequency Emission Mechanism

The origin of the low-frequency spectral peaks observed in the DAS dataset (~77 Hz and 135 Hz) remains uncertain; however, their systematic dependence on flow rate and burial depth suggests a coupling mechanism between the migrating gas and the surrounding sediment. Comparable low-frequency pressure pulses have been documented in natural systems such as the hydrothermal vents of Yellowstone Lake, where brief (1–40 Hz) pressure pulses caused by turbulent flow in venting fluids were interpreted to trigger the formation of CO_2_ bubbles within gas-saturated pore water [31]. Each bubble event displayed a sharp, high-frequency onset associated with the opening of the cavity, followed by lower-frequency oscillations corresponding to the free vibration of the bubble [31]. Although the frequencies observed in our laboratory experiments are higher—partly due to geometric and boundary-scale effects—the overall temporal pattern, characterized by an initial amplitude spike followed by lower-frequency ringing, is consistent with this process.

Across experiments of the primary injection range 100–1000 mL min^−1^, each bubble cluster event exhibited a pronounced amplitude spike preceding the oscillatory bubble waveform recorded by DAS. These spikes were absent from baseline acquisitions and do not coincide with electrical noise (60 Hz and 120 Hz). Therefore, we hypothesize that these spikes are related to the bubble-emergence process rather than instrumental or electrical noise. We interpret the onset pulse as a transient strain response caused by the rapid deformation of sediment grains when a trapped gas pocket breaches the sand–water interface. The subsequent lower-frequency oscillation likely represents the elastic rebound of the surrounding medium or the initial expansion of the bubble itself. While this differs from the hydro-acoustic mechanism described by [31], which emphasized fluid-pressure instabilities as the bubble-nucleation trigger, both cases demonstrate that small, rapid perturbations can generate coupled high- and low-frequency acoustic components in gas-saturated systems. However, it is also possible that the low-frequency peaks could arise from mechanical coupling or resonance within the injection hose and fittings, particularly given their proximity to the fiber coil. The attenuation of these peaks with increasing burial depth and their amplification with flow rate are consistent with a sediment-coupled contribution to the low-frequency response. These observations alone, however, cannot uniquely distinguish sediment-mediated deformation from source-excited mechanical or boundary resonances.

At the highest injection rates (≥700 mL min^−1^), where visual observations indicated the formation of stable bubble chimneys via stationary bubble emergence, the amplitude spectra display a diminished high-frequency pulse and a broad, less distinct low-frequency component. This behavior may result either from constructive interference caused by the rapid emergence of multiple bubbles within the chimney, which smears the impulsive component, or from the continuous venting of gas from a fixed location that delays or suppresses elastic rebound of the sediment. The latter mechanism—progressive loss of sediment rebound due to sustained pore dilation—is consistent with the development of quasi-steady conduits that remain open under high-flux conditions. Thus, the low-frequency peaks likely reflect a combination of pressure-pulse generation at bubble emergence and transient strain associated with sediment relaxation, both of which diminish as the system transitions to continuous chimney flow.

The frequency dependence, depth attenuation, and correspondence with visual observations are consistent with transient sediment deformation contributing to the low-frequency energy recorded by DAS; however, the present experimental configuration does not uniquely establish this mechanism. Further experiments would be necessary to isolate mechanical coupling effects and quantify the relative contributions of sediment strain, fluid-pressure fluctuations, and experimental-system resonances.

### 5.3. DAS as a Monitoring Tool for Marine GCS

Although hydrophones are effective for detecting bubble acoustics in the water column, their point-based measurements limit spatial coverage. Conversely, DAS facilitates the simultaneous detection of bubble oscillations and sediment-coupled signals across extended spatial regions. Therefore, DAS demonstrates a compelling advantage for long-term monitoring of CCS sites, providing comparable detection capabilities with broader spatial applicability.

The template matching results elucidate both the potential and limitations of DAS-based detection of bubble-related acoustic signatures. The observed overmatching of Type I templates reflects the relatively broadband, high-energy, and repeatable oscillatory signature of bubbles in free fluid, which produces more matches but also reduces selectivity when simple cross-correlation thresholds are applied. In contrast, the more selective but less temporally aligned detections observed for waveform Types II–IV likely reflect increased waveform variability associated with bubble–sediment and interface-controlled processes, where local coupling conditions, scattering, and attenuation alter the emitted acoustic signatures. This demonstrates an inherent tradeoff between match occurrence and selectivity when applying fixed-threshold template matching to DAS data, particularly in seafloor environments where leakage signals transition between free fluid and interface-dominated regimes. Importantly, the use of a single cross-correlation threshold across all waveform types avoids bias introduced by threshold tuning and provides a consistent basis for comparing template-matching behavior across a range of leakage conditions. For CCS applications, this suggests that DAS-based monitoring strategies may benefit from multi-template or adaptive detection approaches that account for evolving leakage mechanisms, rather than relying solely on canonical free-bubble signatures.

Furthermore, the observed behavior of template matching reveals that simple cross-correlation is more appropriately employed as an exploratory screening tool than as a definitive classifier of leakage mechanisms. The pronounced overmatching of Type I templates indicates lower selectivity for Type I waveforms under the fixed correlation threshold, while the diminished alignment for Types II–IV reflects the increased waveform variability linked to sediment interactions and interface effects. Consequently, in practical monitoring scenarios, the effectiveness of template matching would be enhanced through integration with complementary indicators such as frequency-band persistence, burial-depth dependence, and temporal recurrence, rather than relying on it as a sole detection criterion. Quantitative detector-performance metrics were not evaluated because bubble-release times were not independently cataloged.

In addition to transient detection and template-based screening, passive acoustic monitoring has been used in prior studies to estimate bubble size distributions and gas flux through spectral inversion of time-averaged acoustic data. In this approach, the acoustic spectrum of a seep is interpreted as the combined resonance response of many bubbles oscillating simultaneously, with each bubble contributing energy at a frequency determined by its size through the Minnaert relation. Because resonance frequency scales inversely with bubble radius, the measured spectral energy distribution can be inverted to recover a bubble size distribution, which may then be integrated to estimate volumetric gas flux [17,19,32]. Rather than relying on the identification and counting of individual bubble transients, this approach infers leakage rate from persistent frequency-band energy associated with bubble resonance. It has been shown to be particularly effective under moderate to high flux conditions, where individual bubble transients overlap and cannot be reliably isolated, allowing flux estimation from sustained spectral energy instead of discrete bubble counting [17,32].

The present study does not implement spectral inversion for quantitative flux estimation. However, the detection of resonance-associated energy within DAS recordings demonstrates that distributed fiber measurements capture the same physical bubble oscillation processes that form the basis of hydrophone-based inversion methods. As such, DAS measurements may provide a spatially extensive dataset that could, with appropriate calibration and transfer-function modeling, support spectral-based leakage quantification for CCS monitoring applications.

### 5.4. Limitations of Controlled Testing Environment

While the controlled laboratory environment enabled precise testing of DAS sensitivity to bubble acoustics, several limitations constrain the direct transferability of these findings to field conditions. First, the maximum detection distance from the fiber remained unconstrained due to the geometry required to accommodate the fiber length to image acoustic waveforms. Coiling the fiber near the bubble source ensured strong signal coupling but restricted sensitivity assessment as a function of increasing standoff distance. Consequently, the detection performance observed in this experiment represents a favorable near-source configuration. This coiled geometry also obscured the directional component of the bubbles as they migrated through the unconsolidated sediment, thus limiting the ability to inform optimal fiber placement strategies for field deployments where bubbles may be meters to hundreds of meters from the sensing array. Because fiber orientation continuously varies around the coil, the measured amplitudes may differ from those obtained using a straight field-deployed cable with a fixed orientation relative to the source. A separate consideration is the 0.8168 m gauge length used in this experiment, which averages the measured response over a shorter section of fiber than may be used in some field DAS deployments. Longer gauge lengths may smooth spatially localized bubble responses and alter their apparent amplitude and spatial extent [27]. Because gauge length was not varied in this study, its influence on bubble detectability and localization was not evaluated. Future experiments incorporating increasing source-fiber offsets, a straight or field-representative cable geometry, and gauge lengths representative of field deployments would be required to quantify attenuation, directional sensitivity, spatial detectability, and practical detection range.

An additional constraint arises from the tank geometry, which inherently limited bubble migration pathways. Unlike in natural marine sediments, where heterogeneous permeability and stratigraphy influence bubble transport, the experimental configuration restricted bubble ascent to essentially vertical pathways. This simplification does not reliably indicate the complexity of gas migration through anisotropic formations. The confined tank geometry may also have influenced the recorded acoustic response through boundary reflections or resonances. Although the tank walls were lined with bubble wrap to reduce reflections and baseline measurements were acquired prior to injection, the acoustic transfer function of the tank was not independently characterized. Therefore, source-excited tank or experimental apparatus resonances cannot be completely excluded as contributors to the observed spectral features, and the absolute frequencies and spectral amplitudes measured in this laboratory investigation should not be assumed to transfer directly to field conditions. Dedicated impulse-response measurements or experiments using multiple source positions would be required to quantify these boundary effects.

Another limitation is the absence of the ambient noise characteristic of natural shallow marine environments. In these environments, mid-frequency acoustic energy from biological sources, small-vessel activity, and infrastructure-related flow noise may overlap with the frequency band associated with bubble oscillations, thereby possibly obscuring or interfering with bubble detection. Controlled field deployments in lakes or nearshore marine sites will be essential for evaluating the robustness of DAS for bubble monitoring in the natural environment. Finally, the controlled 30 s injections were designed to isolate the DAS response to known gas flow conditions rather than reproduce the temporal duration of a field leak; consequently, potential long-term evolution of sediment migration pathways was not evaluated in this study.

The results presented here demonstrate that DAS can resolve bubble resonance frequencies and low-frequency sediment responses associated with gas migration, establishing a basis for its application in leakage monitoring at GCS sites. While absolute amplitudes and characteristic frequencies observed for N_2_ in the laboratory are not expected to transfer directly to CO_2_ leakage or field settings, the qualitative trends identified in this study, including burial depth attenuation, response-amplitude differences between free fluid and sediment-coupled signals, and the persistence of bubble-related frequency bands, provide a basis for evaluating DAS responses under field conditions. These trends are consistent with coupling mechanisms between gas migration and sediment response. Our findings indicate that DAS may establish the presence of leakage and the conditions governing bubble migration, supporting its potential as a scalable monitoring technology for shallow marine environments.

## Figures and Tables

**Table 1:** Experimental N_2_ injection rates and their corresponding CO_2_-equivalent rates. CO_2_-equivalent values were computed by scaling the measured N_2_ volumetric injection rates (mL min^−1^) by the ratio of near-surface gas densities ($\rho_{{\mathrm{CO}}_{2}}=1.98$ kg m^−3^; $\rho_{N_{2}}=1.25$ kg m^−3^), such that the reported CO_2_ rate represents the volumetric flux that would produce an equivalent mass leakage of CO_2_. The percentage column expresses the corresponding equivalent annual CO_2_ mass leakage relative to a nominal 1 Mt yr^−1^ geologic storage injection rate (e.g., Sleipner).

N_2_ Injection Rate (mL min^−1^)	CO_2_-Equivalent Volumetric Rate (mL min^−1^)	Percent of 1 Mt yr^−1^ CCS Injection ($\times 10^{-5}$)
100	63.1	0.657
200	126.3	1.314
300	189.4	1.971
400	252.5	2.628
500	315.7	3.285
600	378.8	3.942
700	441.9	4.599
800	505.1	5.256
900	568.2	5.913
1000	631.3	6.570

## Data Availability

The original data presented in the study are openly available in Zenodo at https://doi.org/10.5281/zenodo.21889934.

## Supplementary Materials

The following supporting information can be downloaded at https://www.mdpi.com/article/10.3390/s26185796/s1, Figure S1: Injection line pressure variations during N_2_ bubble experiments. The plotted time series represent a 200 s window from each experiment and illustrate transient pressure fluctuations associated with gas accumulation and episodic bubble release at the injector outlet. Lower flow rates exhibit larger intermittent pressure excursions, whereas higher flow rates generally display more stable pressure behavior indicative of increasingly continuous gas release. Figure S2: Effect of N_2_ injection flow rate on signal-to-noise ratio (SNR) of bubble-generated acoustic signals. DAS measurements acquired in the fluid, shallow sand burial, and deep sand burial configurations are compared to the hydrophone response across flow rates ranging from 100 to 1000 mL min^−1^. The hydrophone response exhibits lower SNR values than all DAS configurations across the tested flow rates, while the DAS responses show higher SNR values that vary with cable burial depth.

## Abbreviations

The following abbreviations are used in this manuscript:

DAS	Distributed Acoustic Sensing
CCS	Carbon Capture and Storage
GCS	Geologic Carbon Storage
IU	Interrogator Unit
scCO_2_	Supercritical CO_2_
FFT	Fast Fourier Transform
RMS	Root Mean Square
SNR	Signal-to-noise Ratio
