# Supplementary material for: Detecting Seafloor Gas Leakage from Geologic Carbon Storage Sites: A Laboratory-Scale Distributed Acoustic Sensing Evaluation
Source: Sensors (Basel). 2026 Sep 12;26(18):5796. doi: 10.3390/s26185796 (PMC13611313; doi:10.3390/s26185796)
Supplement: Supplementary file 1 [file sensors-26-05796-s001.zip › S1_pressure.pdf]

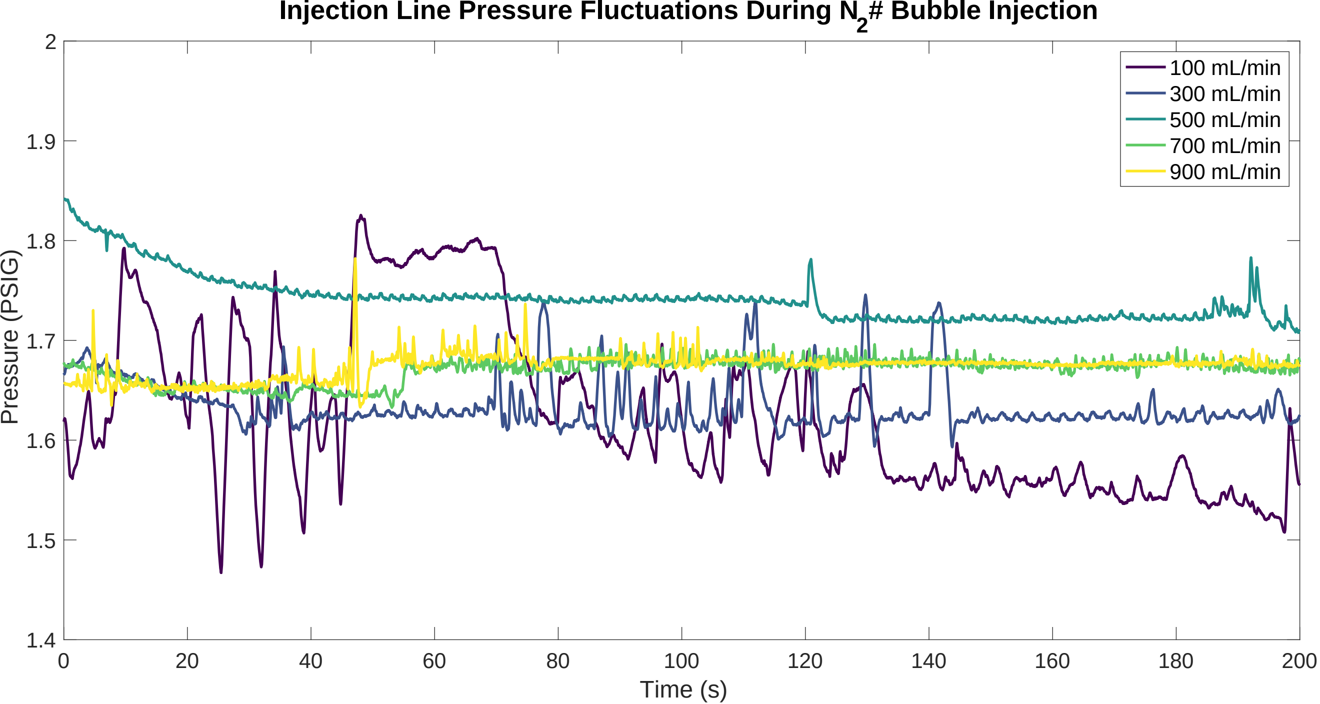
Injection Line Pressure Fluctuations During N2# Bubble Injection
100 mL/min
300 mL/min
500 mL/min
700 mL/min
900 mL/min
Pressure (PSIG)
Time (s)
2
1.9
1.8
1.7
1.6
1.5
1.4
0
20
40
60
80
100
120
140
160
180
200
