# Supplementary material for: Detecting Seafloor Gas Leakage from Geologic Carbon Storage Sites: A Laboratory-Scale Distributed Acoustic Sensing Evaluation
Source: Sensors (Basel). 2026 Sep 12;26(18):5796. doi: 10.3390/s26185796 (PMC13611313; doi:10.3390/s26185796)
Supplement: Supplementary file 1 [file sensors-26-05796-s001.zip › S2_SNR.pdf]

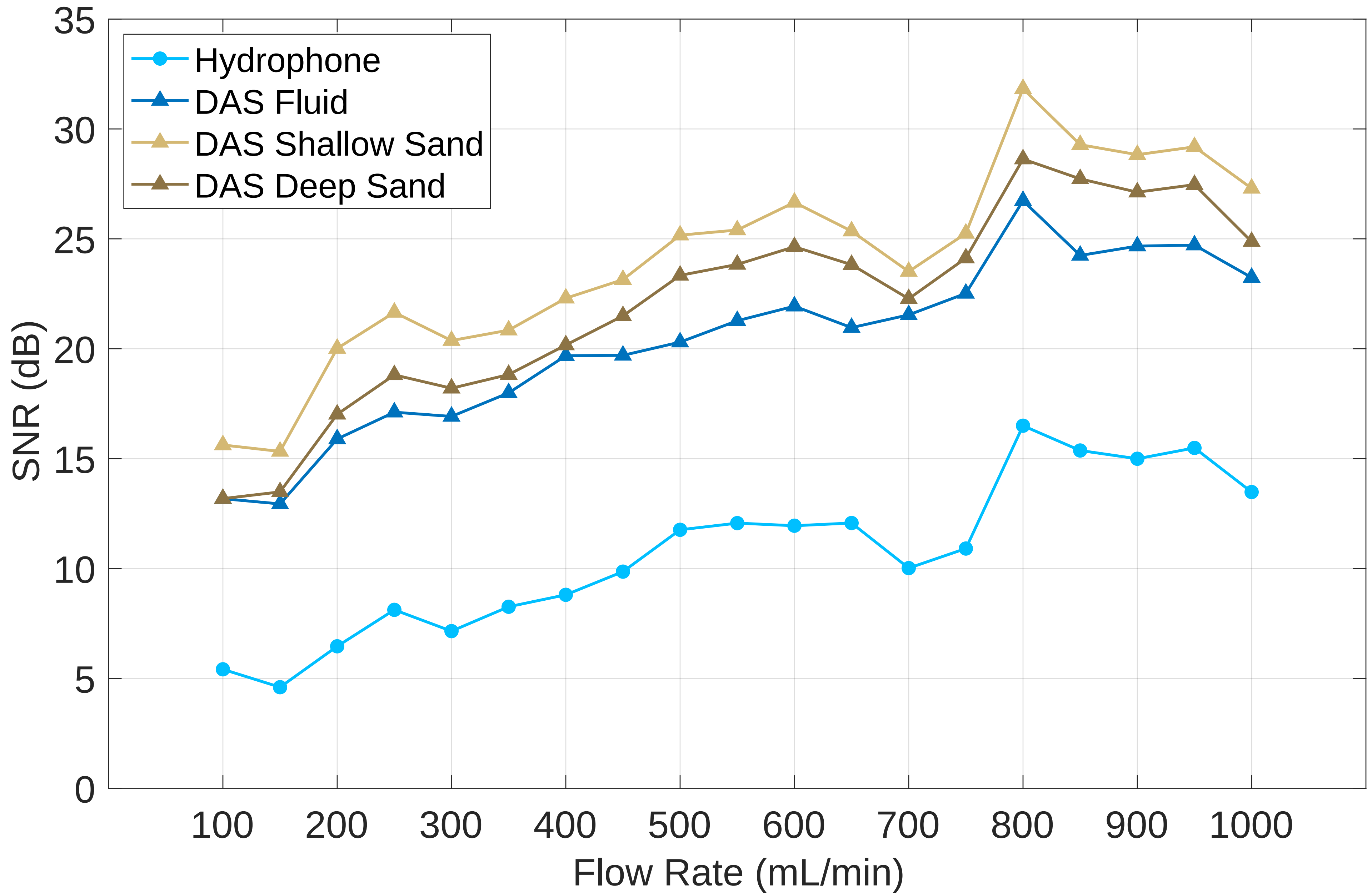
Hydrophone
DAS Fluid
DAS Shallow Sand
DAS Deep Sand
SNR (dB)
0
5
10
15
20
25
30
35
100
200
300
400
500
600
700
800
900
1000
Flow Rate (mL/min)
